# Changes in Internet Search Term Popularity in Elder Mistreatment (2018-2023): Infodemiology Study of Google Trends Data

**DOI:** 10.2196/83797

**Published:** 2026-04-21

**Authors:** Nicholas Sevey, Melvin Livingston, Kristin Lees-Haggerty, Olanike Ojelabi, Randi Campetti, Jason Burnett, Carolyn E Z Pickering, David Hancock, Rachit Sabharwal, Michael Brad Cannell

**Affiliations:** 1 Department of Internal Medicine, University of Texas Southwestern Medical Center, Dallas, TX, United States; 2 Department of Pediatrics, University of Texas Southwestern Medical Center, Dallas, TX, United States; 3 Department of Behavioral Sciences and Health Education, Rollins School of Public Health, Emory University, Atlanta, GA, United States; 4 Education Development Center, Inc., Waltham, MA, United States; 5 Center for Social Policy, University of Massachusetts Boston, Boston, MA; 6 Joan and Stanford Alexander Division of Geriatric and Palliative Medicine, McGovern Medical School, University of Texas Health Science Center at Houston, Houston, TX, United States; 7 Cizik Nursing Research Institute, Department of Research, Cizik School of Nursing, University of Texas Health Science Center at Houston, Houston, TX, United States; 8 Division of Geriatric Emergency Medicine, Department of Emergency Medicine, Weill Cornell Medical College, New York, NY, United States; 9 School of Public Health, University of Texas Health Science Center at Houston, Houston, TX, United States; 10 Center for Neurodegenerative Disease, Department of Health Sciences and Public Health, Harris College of Nursing and Health Sciences, Texas Christian University, 2800 W. Bowie Street, Ste. 2101, Fort Worth, TX, 76109, United States, 1 8172571426

**Keywords:** elder abuse, elder maltreatment, elder neglect, Google Trends, abuse reporting, reports

## Abstract

**Background:**

Elder mistreatment (EM) is a significant public health problem that is frequently underdetected and underreported. Insufficient public recognition and engagement have been hypothesized as one contributor to this underreporting; however, few data sources exist to quantify public awareness or engagement with EM at the population level.

**Objective:**

This study examined relative internet search interest in EM compared with other forms of abuse (child abuse and domestic violence) in the United States using Google Trends data.

**Methods:**

We analyzed Google Trends data to compare the Relative Search Index (RSI) for the terms “elder abuse,” “child abuse,” and “domestic violence” in the United States from December 2018 to December 2023. RSI values reflect normalized search activity relative to the maximum search volume within the specified period.

**Results:**

Mean RSI values for “elder abuse” were substantially lower than those for “child abuse” (11.35 vs 50.21) and “domestic violence” (6.96 vs 63.50). Ad hoc tests for stationarity indicated that RSI values for all 3 terms remained stable over the 5-year study period. During Elder Abuse Awareness Month, RSI for “elder abuse” increased relative to “child abuse” and “domestic violence” by 8.3 and 5.7 points, respectively, compared with other months of the year.

**Conclusions:**

Relative search interest in elder abuse appears to be persistently lower than that for child abuse and domestic violence, despite modest increases during Elder Abuse Awareness Month. Although Google Trends does not provide a validated measure of public awareness, search-based metrics such as RSI may offer a scalable, low-cost complement to traditional data sources for contextualizing public engagement with EM and informing future awareness, detection, and prevention efforts.

## Introduction

Elder mistreatment (EM) is generally defined as an intentional act or failure to act by a caregiver or another person in a relationship involving an expectation of trust that causes or creates a risk of harm to an older adult [1]. In the United States, up to 14% of adults older than 70 years have experienced some form of EM [2]. Most commonly, EM takes the form of neglect, financial abuse, and emotional abuse but can also include physical and sexual abuse, often with dire consequences including increased mortality risks [2-6]. Furthermore, elder financial abuse is estimated to cost billions of dollars per year [7]. This figure is particularly concerning given that financial abuse (similar to many forms of EM) is considered to be grossly underreported, with an estimate of only 1 in 24 cases being reported [7-9].

One hypothesized reason for underreporting includes insufficient public awareness of EM [10]. Prior research consistently characterizes EM as a hidden and underrecognized public health problem, shaped by stigma, dependency, fear of disclosure, and limited institutional detection pathways [5,11,12]. Because EM often occurs in private settings and survivors may be reluctant or unable to seek help, few cases come to the attention of formal service systems [12]. Furthermore, prevention and response efforts are constrained by gaps in public awareness and societal recognition, limiting sustained attention and coordinated intervention [13]. Together, prior literature suggests that low public visibility of EM reflects not only prevalence or legal frameworks but also broader societal and structural barriers to recognition, providing critical context for the comparatively low levels of EM-related Google search activity examined in this study.

Despite ongoing public awareness campaigns [14-16], we are unaware of any nationally representative polls or studies that measure public awareness of EM. This lack of data is a barrier to quantifying current levels of public awareness of EM and evaluating the impact of campaigns, programs, and policies intended to increase public awareness. Although administrative data sources (eg, National Incident-Based Reporting System) might seem suitable for this purpose, existing crime and reporting systems do not comprehensively capture several of the most prevalent forms of EM, such as neglect and emotional or psychological abuse, limiting their usefulness for assessing population-level awareness or incidence. As a novel, *preliminary step* toward overcoming this barrier, we used publicly available data from Google Trends (Google LLC) to explore relative Google search volumes for EM. The objectives of these analyses are to demonstrate the use of Google Trends as a convenient, cost-effective method for establishing and comparing baseline measures of relative search interest (RSI), including in relation to other forms of abuse (ie, child abuse and domestic violence). We also aim to explore the utility of Google Trends to inform future efforts designed to increase public awareness and engagement around EM.

## Methods

We used publicly available data from Google Trends [17] to explore Google search patterns related to EM. Google Trends data are accessed through a web application that allows users to specify 1 or more search terms of interest along with location information and a timeframe of interest. We queried Google Trends for data on the relative volume of Google searches in the United States each week for the term “elder abuse” during a 5-year period from December 2018 to December 2023. The term “elder abuse” was specifically used to reflect its colloquial understanding that encompasses various forms of EM, a term often favored in academia [18]. As a sensitivity analysis, we also examined Google Trends data for the term “elder mistreatment.” RSI values for this term were zero for nearly the entire study period, with the exception of 2 isolated weeks. This pattern suggests minimal public-facing search activity for “elder mistreatment.” Accordingly, we retained “elder abuse” as the primary search term to reflect common public use rather than conceptual equivalence with the broader academic construct of EM.

**Figure 1.**
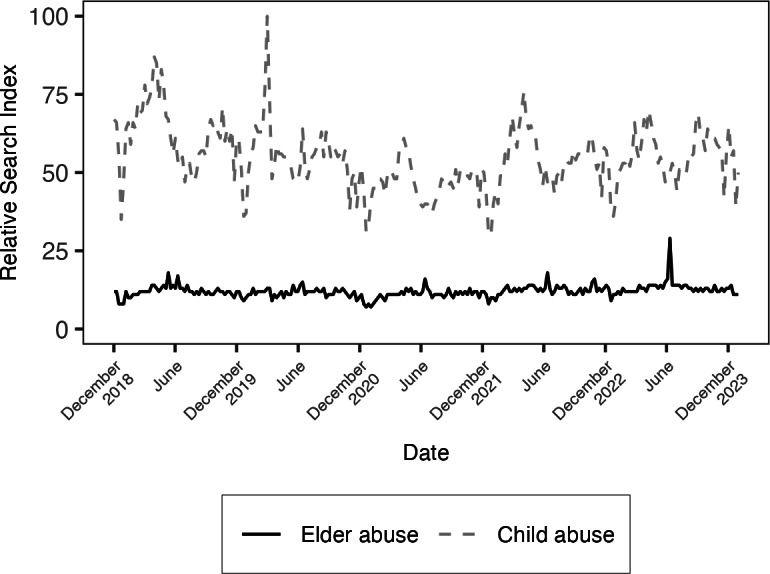
Plot of the Google Trends Relative Search Index for the search terms “elder abuse” and “child abuse” in the United States from December 2018 to December 2023.

**Figure 2.**
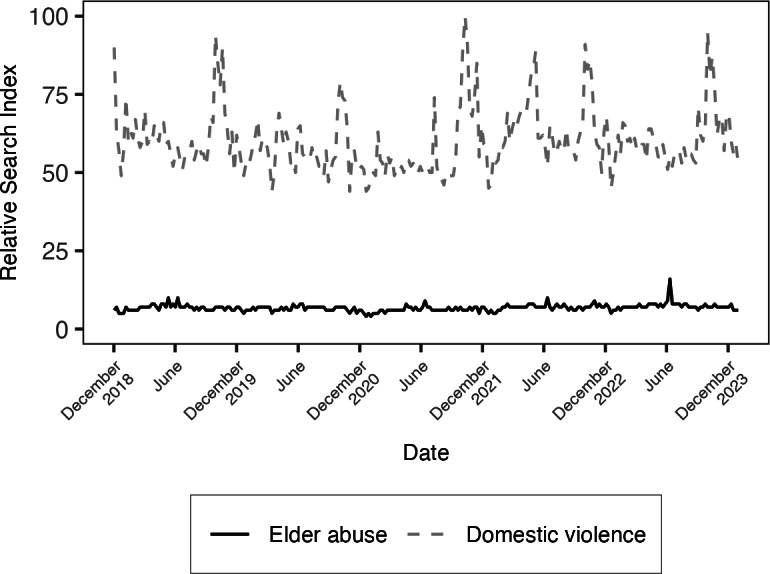
Plot of Google Trends Relative Search Index for the search terms “elder abuse” and “domestic violence” in the United States from December 2018 to December 2023.

For a given Google Trends query, a graph similar to Figures 1 and 2 is generated that shows the relative popularity of one or more search terms in the specified location and timeframe. Each data point on the graph can be downloaded in tabular form for further analysis and reflects the number of searches for the term relative to the total number of Google searches in the same location and timeframe. Importantly, Google Trends queries do not provide the absolute number of searches for each term (eg, 4000 searches for the term “elder abuse” in week X); instead, they return a normalized relative search index (RSI) for each search term, which is an integer value that exists on a scale from 0 to 100, where each data point is divided by the highest point or 100 [19]. In other words, a value of 100 represents the term’s peak popularity within the search period but reveals nothing about total search volumes.

It is also possible to query Google Trends data for multiple search terms simultaneously. Doing so still does not produce any information about the absolute volume of searches for either term, but it does provide information about the relative search volume of the terms, for example, that one search term is twice as popular as another search term for a given place and time. We compared the RSI of “elder abuse” to the RSIs of “child abuse” and “domestic violence” during the same timeframe. The additional context allowed us to draw conclusions about the relative search volume of elder abuse compared to other forms of abuse.

As a further exploratory step, we investigated whether the relative search popularity of elder abuse compared to other forms of abuse varied during periods of heightened awareness about elder abuse, specifically Elder Abuse Awareness Month, which happens every June. For each comparison to elder abuse, we created a long-form data set of the RSIs by week with an indicator for whether the abuse type was elder abuse or another abuse type. We additionally created an indicator for Elder Abuse Awareness month. To estimate the relative change during Elder Abuse Awareness month, we ran generalized estimating equations including the indicators of abuse type, Elder Abuse Awareness Month, their interaction, and controlling for year as a fixed effect. The coefficient for the interaction can be interpreted as the difference in RSI between elder abuse and the comparison abuse type during elder abuse month relative to the difference during other months of the year. All models clustered SEs at the year level to account for potential autocorrelation. Sensitivity analyses logging RSI values found similar results to our primary specification and are not discussed further.

## Results

When comparing elder and child abuse at the national level, the search term “elder abuse” had a mean RSI of 11.35 (SD 1.91) with a peak RSI of 26.00, whereas “child abuse” had a mean RSI of 50.21 (SD 10.14) with a peak popularity of 100.00 (Figure 1). When comparing elder abuse and domestic violence, the search term “elder abuse” had a mean RSI of 6.96 (SD 1.15) with a peak popularity of 16.00, whereas “domestic violence” had a mean RSI of 63.51 (SD 8.96) with a peak popularity of 100.00 (Figure 2). While short-term fluctuations were observed, ad hoc tests for stationarity indicate that baseline search interest trends were stable throughout the 5-year study period.

The results of our exploratory analyses found statistically significant changes in the difference in RSI by abuse type during Elder Abuse Awareness Month. Compared to other months of the year, the RSI for “elder abuse” increased from an estimated 11.2 points to 13.23 points, while the RSI for “child abuse” fell from an estimated 50.7 points to 44.5 points, resulting in a net increase of 8.3 points for the RSI of “elder abuse” during Elder Abuse Awareness month (β=8.29, 95% CI 5.70-10.88; Z=6.27; *P*<.001). A similar pattern was observed comparing “elder abuse” to “domestic violence.” Compared to other months of the year, the RSI for “elder abuse” increased from an estimated 6.9 points to 8.1 points, while the RSI for “domestic violence” fell from an estimated 63.9 points to 59.4 points, resulting in a net increase of 5.7 points for the RSI of “elder abuse” during Elder Abuse Awareness month (β=5.72, 95% CI 2.55-8.90; Z=3.54; *P*<.001). Notably, these changes were driven largely by a reduction in the relative search volumes for “child abuse” and “domestic violence,” and not by the relatively small increase in RSI for “elder abuse.” In both cases, the RSI for “elder abuse” remained substantially lower than its comparators.

## Discussion

### Principal Findings

EM is an important and pervasive public health problem that is exacerbated by chronic underdetection and underreporting. Insufficient public awareness of EM is one hypothesized reason for its underdetection and underreporting [2]. Our results provide preliminary evidence of the following: (1) Google Trends may feasibly be used to measure the RSI of EM, (2) the RSI for EM is lower than it is for child abuse and domestic violence, (3) the RSI of EM remained relatively unchanged between December 2018 and December 2023, and (4) the RSI for “elder mistreatment” was essentially zero during the study period.

### Measuring Public Awareness May Be Feasible With Google Trends

Currently, there are no nationally representative polls or studies that measure public awareness of EM. This gap perpetuates barriers in assessing efforts to increase public awareness of EM and ultimately its detection, intervention, and prevention. Ideally, public awareness of EM would be rigorously measured longitudinally, at the state and national levels, in a population-representative way. However, such studies typically require significant planning, resources, and political prioritization. In the meantime, other less-rigorous measures of public awareness can serve as an important stopgap. To the best of our knowledge, this is the first study that objectively compares the RSI of EM to the RSI of other forms of abuse including child abuse and domestic violence, albeit with some important limitations.

While the technical definition of what Google Trends data measure (ie, the RSI) is clearly defined, we recognize the challenge of identifying a single term—such as “public awareness”—that accurately captures its practical meaning, particularly when attempting to distinguish “public awareness” from related constructs such as “public interest.” To the best of our knowledge, empirically robust methodological evidence does not currently exist to support the claim that Google Trends RSI is a valid and reliable proxy measure of public awareness. Nonetheless, a growing number of published studies have used Google Trends data in this way [20-22]. We posit that Google Trends RSI likely reflects search activity among individuals who possess some awareness of EM *and* have sufficient interest and access to seek information using Google. However, more rigorous research, which is beyond the scope of this study, is needed to empirically evaluate this interpretation.

If one is willing to accept that Google search volume is a useful proxy measure of —or at least closely associated with—public awareness, then this study provides an example of how the public awareness of EM may feasibly be estimated by the relative popularity of related search terms over time. In fact, such methods are increasingly represented in the literature as a measure of public awareness for topics ranging from concussions in Australia following the National Brain Injury Awareness Week to rheumatic disease in relation to high-profile diagnoses among celebrities and the use of glucagon-like peptide 1 agonists such as “Ozempic” for cosmetic weight loss [23-25].

### The RSI for EM Is Lower Than That for Other Forms of Abuse

In this analysis, the RSI for EM was lower than for other forms of abuse. Despite the fact that estimates of the past year prevalence of child abuse are comparable to EM (14% for child abuse [26] and 10% for EM [23], about 1.4 times higher), the public used the search term “child abuse” approximately 4 times as often as “elder abuse” during the 5-year study period. Similarly, although the estimated past year prevalence of domestic violence (7.3% of women and 6.8% of men [27]) is roughly 1.4 times lower than the past year prevalence of EM, the search term “domestic violence” was used approximately 9 times as often as “elder abuse” during the 5-year study period, as measured by Google Trends RSI. Extreme caution is warranted in interpreting these comparisons. The accuracy and comparability of these past year prevalence estimates are questionable for a number of reasons; however, the magnitude of the discrepancy between the underlying prevalence of these abuse types and their Google search volumes is consistent with the results we would expect to find if the public were indeed less aware of EM than other forms of abuse. That said, baseline differences between EM and other forms of abuse may influence public awareness and search term popularity and are worthy of inclusion in future studies exploring public awareness of EM.

### RSI Remained Relatively Unchanged Over Time

The results of this study also suggest that, aside from a modest relative increase during the World Elder Abuse Awareness Month, the relative popularity of Google searches for “elder abuse” remained relatively unchanged over the study period. However, we did observe a stable, non-zero level of public awareness, which could serve as a baseline to assess future public health interventions, including changes in EM policies and the implementation of public awareness campaigns. Although Google Trends data alone do not provide information about the causes of this trend, its stability suggests that short-term or sporadic efforts may not be sufficient to sustain meaningful change, as acute events such as increased media coverage may only produce superficial short-lived spikes in searches. Instead, public policies backing sustained efforts over an extended period may be needed to shift societal attitudes about aging and increase awareness of EM. That said, RSI is a single crude measure that reveals nothing about the intentions behind an individual’s search nor about their level of awareness. Future research should further explore event-driven analyses (eg, news stories involving celebrities, World Elder Abuse Awareness Day, and the US Postal Inspection Service campaign) to investigate how the public responds to these campaigns and events reflected by specific keyword searches (eg, financial fraud or survivor services). Analyses should also seek to determine if these increases are sustained over time. For example, future interrupted time series analyses could provide valuable insight into the short- and long-term effects of these campaigns.

Similarly, the near absence of search activity for the term “elder mistreatment,” contrasted with sustained search interest in “elder abuse,” may reflect a disconnect between academic terminology and public-facing language. Although “elder mistreatment” is widely used in research and policy contexts to capture a broader range of harmful experiences, the public may be more familiar with or responsive to the term “elder abuse.” This observation has potential implications for the design and evaluation of public awareness campaigns, suggesting that efforts to increase recognition and engagement may benefit from careful consideration of terminology. Future research could examine how language choice influences public attention, search behavior, and engagement with prevention and reporting resources.

### Limitations

Using normalized, relative search term popularity (ie, RSI) derived from Google Trends represents a novel approach for examining patterns related to EM. However, this approach has notable limitations. A clear, practical definition of what RSI measures, backed by empirical evidence, does not currently exist. Framing RSI as a more easily understood construct (eg, public awareness) is appealing and could improve the practical value of studies such as this one; however, generating the empirical evidence needed to support such a framing is beyond the scope of this study. Furthermore, Google Trends cannot tell us about people who are aware of EM but not interested, nor those who are aware and interested but lack digital literacy or internet access. It also cannot identify those who are unaware of EM, regardless of whether they might become interested if awareness were increased. Future studies that attempt to tease apart the nuances of what Google Trends data are measuring in the context of family violence may prove to be valuable to the field.

A second major limitation is that the RSI provided by Google Trends represents the relative popularity of a search term in relation to the total number of searches within a specified time and location; absolute numbers of searches are not available. Furthermore, the RSI does not include search term variations such as misspellings, synonyms, or translations into other languages, nor does it address overlap in colloquial understandings among these terms, for example, in cases where one may categorize EM as a form of domestic violence and search for that term instead. Future studies would benefit from broadening the number of related terms to better capture these contributory permutations. Moreover, integrating complementary methods of measuring public awareness of EM (social media chatter, signal detection, etc) would provide an even greater insight into how the public expresses both awareness and interest in EM.

Additionally, our comparisons of “elder abuse” to “child abuse” and “domestic violence” used mean RSIs over a 5-year period. Short-lived spikes in popularity could potentially coincide with discrete efforts to increase public awareness of EM (eg, World Elder Abuse Awareness Day), significant current events associated with increases in EM (eg, the COVID-19 pandemic), or headline news stories about allegations of EM perpetrated against celebrities such as Stan Lee, Mickey Rooney, Brooke Astor, and Richard Simmons. However, despite observing periodic spikes in popularity, we found no sustained changes for any search terms of interest throughout the study period as measured by tests for stationarity. This suggests that any potential influences on EM awareness during the study period were likely transient.

Despite these limitations, there is growing evidence that Google Trends data have the potential to serve as a useful measure of public awareness in some circumstances [28,29].

### Public Health Implications

EM is a significant public health problem that remains underdetected and underreported, in part due to limited public recognition and engagement. Using Google Trends data, this study demonstrates a persistent disparity in relative search interest between EM and other forms of abuse such as child abuse and domestic violence. This methodological approach is novel in its direct comparison of internet search behavior across different forms of abuse. By directly comparing internet search behavior across these forms of abuse, this work introduces a novel, scalable approach for contextualizing public engagement with EM over time and geographic location.

Although Google Trends does not provide a validated measure of public awareness, search-based metrics such as RSI may offer a low-cost and widely accessible complement to traditional surveillance systems, particularly in settings where population-representative measures are unavailable. Over time, such approaches could help inform the targeting and evaluation of public awareness campaigns, generate hypotheses about gaps in engagement, and support broader efforts aimed at improving detection, prevention, and response to EM.

## Abbreviations

EM: elder mistreatment
RSI: relative search index
